# Development of a Follow-Up Measure to Ensure Complete Screening for Colorectal Cancer

**DOI:** 10.1001/jamanetworkopen.2024.2693

**Published:** 2024-03-25

**Authors:** Elizabeth L. Ciemins, Jeff T. Mohl, Carlos A. Moreno, Francis Colangelo, Robert A. Smith, Mary Barton

**Affiliations:** 1 Research and Analytics, American Medical Group Association, Alexandria, Virginia; 2 Now with Albany Medical College; 3 Allegheny Health Network, Pittsburgh, Pennsylvania; 4 Center for Cancer Screening, American Cancer Society, Atlanta, Georgia; 5 National Committee for Quality Assurance, Washington, DC

## Abstract

**Importance:**

The current quality performance measure for colorectal cancer (CRC) screening is limited to initial screening. Despite low rates, there is no measure for appropriate follow-up with colonoscopy after receipt of an abnormal result of a stool-based screening test (SBT) for CRC. A quality performance measure is needed.

**Objective:**

To develop and test a quality performance measure for follow-up colonoscopy within 6 months of an abnormal result of an SBT for CRC.

**Design, Setting, and Participants:**

This retrospective quality improvement study examined data from January 1, 2016, to December 31, 2020, with 2018 plus 6 months of follow-up as the primary measurement period to verify performance rates, specify a potential measure, and test for validity, reliability, and feasibility. The Optum Labs Data Warehouse (OLDW), a deidentified database of health care claims and clinical data, was accessed. The OLDW contains longitudinal health information on enrollees and patients, representing a diverse mixture of ages and geographic regions across the US. For the database study, adults from 38 health care organizations (HCOs) aged 50 to 75 years who completed an initial CRC SBT with an abnormal result were observed to determine follow-up colonoscopy rates within 6 months. Rates were stratified by race, ethnicity, sex, insurance, and test modality. Three HCOs participated in the feasibility field testing. Data were analyzed from June 1, 2022, to May 31, 2023.

**Main Outcome and Measures:**

The primary outcome consisted of follow-up colonoscopy rates following an abnormal SBT result for CRC. Reliability statistics were also calculated across HCOs, race, ethnicity, and measurement year.

**Results:**

Among 20 581 adults (48.6% men and 51.4% women; 307 [1.5%] Asian, 492 [7.2%] Black, 644 [3.1%] Hispanic, and 17 705 [86.0%] White; mean [SD] age, 63.6 [7.1] years) in 38 health systems, 47.9% had a follow-up colonoscopy following an abnormal SBT result for CRC within 6 months. There was significant variation between HCOs. Notably, significantly fewer Black patients (37.1% [95% CI, 34.6%-39.5%]) and patients with Medicare (49.2% [95% CI, 47.7%-50.6%]) or Medicaid (39.2% [95% CI, 36.3%-42.1%]) insurance received a follow-up colonoscopy. A quality performance measure that tracks rates of follow-up within 6 months of an abnormal SBT result was observed to be feasible, valid, and reliable, with a median reliability statistic between HCOs of 94.5% (range, 74.3%-99.7%).

**Conclusions and Relevance:**

The findings of this observational study of 20 581 adults suggest that a measure of follow-up colonoscopy within defined periods after an abnormal result of an SBT test for CRC is warranted based on low current performance rates and would be feasible to collect by health systems and produce valid, reliable results.

## Key Points

**Question:**

What are the supporting evidence, feasibility, reliability, and validity of a quality performance measure on follow-up colonoscopy after an abnormal result of a stool-based screening test (SBT) for colorectal cancer (CRC)?

**Findings:**

In this quality improvement study including 20 581 adults at 38 health care organizations, 48% of patients received a colonoscopy within 6 months after an abnormal result of an SBT for CRC. A quality measure that tracks follow-up rates within 6 months of SBT is feasible, valid, and reliable.

**Meaning:**

These findings suggest that a measure on follow-up colonoscopy after an abnormal result of an SBT for CRC is warranted based on low current performance rates and high feasibility, validity, and measurement reliability.

## Teaser

This quality improvement study describes the measure development, testing process, and results for a screening completion measure among patients with positive results of a stool-based screening test for colorectal cancer (CRC).

## Introduction

Colorectal cancer (CRC) is the third leading cause of cancer deaths in men and women in the US, and it is estimated that there will be approximately 53 000 deaths due to CRC in 2023.^1^ Screening for CRC is widely recommended^2,3,4,5^ because, when identified early, CRC is one of the most treatable forms of cancer, with a 5-year survival rate of 90%.^6,7^ In contrast, the 5-year survival rate among those diagnosed with late-stage disease is only 14%.^7^ Currently, only 33% of CRCs are diagnosed at the earliest stage.^1^ Individuals with an abnormal result of a stool-based screening test (SBT) must receive timely follow-up with a colonoscopy because patients experience increased negative clinical outcomes, such as later-stage diagnosis, when follow-up after an abnormal SBT result is delayed by more than 6 to 12 months.^8,9,10^ Diagnosis at an early stage will reduce neoplastic progression of disease^8,9,10^ and therefore increase the possibility of curative treatment.^9^ It follows that early diagnosis may also decrease downstream complications, improve outcomes, and minimize costs to the patient and the health care system.^11^ Further, early diagnosis helps reduce racial and ethnic disparities in outcomes: when CRC is diagnosed at a localized stage, survival rates are comparable across racial and ethnic groups.^12^

Patients and health systems are increasingly using SBTs for convenience and patient preference^13,14^ and to maximize population-level screening rates without a substantial increase in costs.^15^ However, timely (within 6 months) follow-up after an abnormal SBT result is suboptimal.^16,17,18,19^ In a study using the Optum Labs Data Warehouse (OLDW), follow-up rates at 3 and 6 months were low, at 43% and 51%, respectively.^20^ Some health systems have been able to achieve effective follow-up of 85% of patients within 6 months,^21^ but substantial variation exists.^20^

Quality measures can lead to improved performance,^22,23,24,25,26,27^ but the current CRC screening Healthcare Effectiveness Data and Information Set (HEDIS) measure^28^ is incomplete for individuals receiving SBTs for screening. The CRC screening measure is numerator compliant if an SBT is performed but does not account for the result of the test. Only when the SBT result is negative (or normal) is the screening process complete. If the SBT result is positive (or potentially abnormal), a follow-up colonoscopy is required to detect the presence of neoplasia. For these patients, the existing HEDIS measure only captures the first step in the screening process.

To close this gap, we developed and tested an additional measure to track the completion of screening for patients with abnormal SBT results. While the proposed measure would complement the existing HEDIS CRC screening measure used in health plans, we propose this measure for reporting by health systems due to the availability of test results in EHR data. The CRC screening completion measure will assess the rates of timely (within 6 months) follow-up colonoscopy for adults aged 45 to 75 years who completed an initial stool-based CRC screening test (fecal occult blood test, fecal immunochemical test, or multitarget stool DNA) with an abnormal result. A follow-up window of 180 days was supported by studies that demonstrated an increased risk of any stage of CRC when colonoscopies are conducted at least 6 months following an abnormal SBT result.^1,8,9^ The measure is further stratified by race and ethnicity to gauge disparities in CRC screening and follow-up. This addresses the growing trend by quality measure stewards to institute stratification by proxy variables for at-risk populations to address health equity and adjust for patient mix across organizations. As part of the measure development process, we engaged 4 national experts to guide the development and specification of the measure. We then tested the robustness and reliability of the measure using an existing database of electronic health record (EHR) and adjudicated claims data.

The proposed CRC screening completion measure is a novel, innovative measure concept that builds on and addresses an important shortcoming in an existing measure. The proposed measure, combined with the current HEDIS measure, ensures that patients have received a complete screening, which includes a follow-up colonoscopy after an abnormal stool-based test result. This report will describe the measure development and testing process, as well as results, for the CRC screening completion measure*.*

## Methods

This study was deemed exempt from institutional review board approval and the need for informed consent by the Institutional Review Board Affairs Department of WCG because the research was limited to interactions involving educational tests, survey procedures, interview procedures, or observations of public behavior. We followed the Strengthening the Reporting of Observational Studies in Epidemiology (STROBE) and Standards for Quality Improvement Reporting Excellence (SQUIRE) reporting guidelines.

### Data Source

This study primarily used deidentified EHR data—including clinical, demographic, medication, laboratory, and utilization or visit data—from January 1, 2016, to December 31, 2020, available in the OLDW. These data are sourced from more than 50 US health care organizations (HCOs) and have been normalized and standardized into a single database. The OLDW contains longitudinal health information on enrollees and patients, representing a diverse mixture of ages and geographical regions across the US. Adjudicated, deidentified administrative claims data were also accessed for a sensitivity analysis of received services, such as follow-up colonoscopies, provided outside of the index health system where the index CRC screening occurred. Approximately 10% of patients in the OLDW also have claims data, and this overlap was used exclusively in the sensitivity analysis. These claims data include medical and pharmacy claims, laboratory results, and enrollment records for commercial and Medicare Advantage enrollees. Study data were accessed using techniques compliant with the Health Insurance Portability and Accountability Act of 1996.

### Measure Specification

For the study, the measure specified was the percentage of adults aged 50 through 75 years who completed a colonoscopy within 180 days following an abnormal SBT result for CRC within the measurement year. At the time of the study, the guideline-recommended lower age bound was 50 years. The denominator was defined as the number of adults aged 50 through 75 years with an abnormal CRC screening SBT result within the measurement year. The numerator was defined as the number of individuals in the denominator who received a colonoscopy within 180 days of the positive test result. The 180-day follow-up window extended into the subsequent measurement year to account for patients who received an abnormal SBT result in the second 6 months of the measurement year. Patients were excluded if they had a previous CRC diagnosis (*International Classification of Diseases, Ninth Revision* [*ICD-9*], codes 153.8 or 153.9; *International Statistical Classification of Diseases and Related Health Problems, Tenth Revision* [*ICD-10*] codes C18.0 or C18.1); had a history of total colectomy (*ICD-9 Clinical Modification* codes 45.81 or 45.82, *ICD-10 Procedure Classification* codes 0DTE0ZZ or 0DTE4ZZ; *Current Procedural Terminology* [*CPT*] code 44150 or 44151); had a diagnostic SBT (*CPT* code 82271 or 82272); or were receiving palliative or hospice care (*ICD-9* code V66.7, *ICD-10* code Z51.5, *CPT* code 99377 or 99378, or Healthcare Common Procedure Coding System code G0182). Health care organizations with less than 20 eligible index patients and those with less than 100 colonoscopies performed in the measurement year were excluded, leaving 38 HCOs in the analytic dataset. The measure schematic is provided in eFigure 1 in Supplement 1.

### Measure Testing Procedures 

The specified measure performance rates were calculated and stratified by several patient characteristics, including race and ethnicity. One-way analysis of variance (ANOVA) was used when necessary to determine whether measure performance differed across patient strata. Measure performance was also compared across 38 HCOs. A repeated-measures ANOVA was used to test the change in HCO performance over time. To assess reliability, a β-binomial distribution^29^ was fit to quantify the variance within and across strata of interest (HCO, race, ethnicity, and year). The reliability statistic represents the percentage of variance across the population that is due to between-category differences vs variability within a category and is a quantification of how well a measure can distinguish 2 or more strata. Reliability of at least 70% is considered sufficient when comparing groups of individuals.^29^ Two sensitivity analyses were also conducted. The first compared a 180-day follow-up with a shorter follow-up period of 90 days. The second used adjudicated claims data to identify colonoscopies that occurred outside of the health system where the index SBT result was returned.

Feasibility testing included assessments of face validity as determined by the national expert advisors during 6 monthly 1-hour meetings and external feasibility field testing to assess the plausibility of collecting all required data elements for a performance measure and automatically calculating the measure in an EHR system for e-measurement. The National Quality Forum’s feasibility scorecard was used to field test the measure. The scorecard assessed data availability, accuracy, data standards, and work flow related to capturing the data required to calculate the measure by which each data element was scored. Descriptions of the data element domains in the scorecard are provided in eTable 1 in Supplement 1.

### Measure Testing Population or Participants

**Figure 1.**
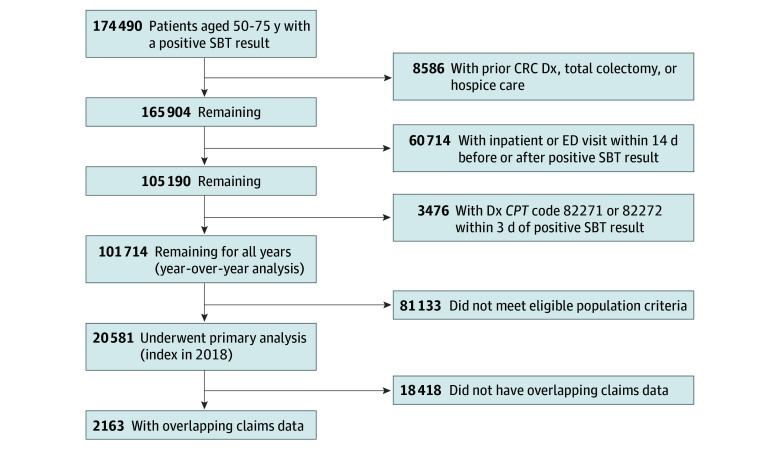
Measure Testing Population Attrition *CPT* indicates *Current Procedural Terminology*; CRC, colorectal cancer; Dx, diagnosis; ED, emergency department; and SBT, stool-based screening test.

The measure testing population included patients who were at average risk and were eligible for stool-based CRC screening at the time of the study. This selection was based on the US Preventive Services Task Force recommendations for CRC screening in 2020 for patients aged 50 to 75 years. In 2021, the US Preventive Services Task Force revised the recommendation to include individuals aged 45 to 75 years, as supported by studies reporting increased rates of CRC among adults aged 45 to 49 years.^1,14^ Because fecal occult blood tests are also used diagnostically to test for blood in the stool unrelated to CRC, particularly in the inpatient and emergency care setting, we excluded test results that were associated with either of these settings (within 14 days of index). We selected 2018 as the primary year for measure evaluation because it was the most recent available year for which follow-up was not affected by the COVID-19 pandemic. Data were available for all years from 2016 through 2020, though this larger dataset was used only for the year-by-year comparisons. An attrition diagram is shown in Figure 1.

### Statistical Analysis

Data were analyzed from June 1, 2022, to May 31, 2023. R software, version 4.1.0 (R Project for Statistical Computing), was used for data processing and analysis. The fitdistrplus package was used to estimate β-binomial distributions. A priori significance levels were set at a 2-sided *P* < .05, and 2-sided tests were used for all comparisons. Uncertainty was quantified using 95% CIs for patient level characteristics or using the IQR when comparing across HCOs.

## Results

### Measure Population Description

Using the eligible population criteria, the EHR-derived population for measure evaluation included 20 581 patients with an abnormal SBT result in the 2018 measurement year. Of these, 2163 had overlapping adjudicated administrative claims data that were used in a sensitivity analysis to examine colonoscopy receipt outside of the index health system. Twenty-two of the HCOs were integrated delivery systems, representing 17 181 patients (83.5%) in the sample. Table 1 provides patient characteristics of EHR-derived and overlapping claims-based populations.

**Table 1.** Patient Characteristics in 2018

Characteristic	Patients^a^	
	EHR dataset (n = 20 581)	Claims dataset (n = 2163)
Index age, mean (SD), y	63.6 (7.1)	64.6 (7.1)
Sex		
Men	10 009 (48.6)	1006 (46.5)
Women	10 572 (51.4)	1157 (53.5)
Race		
Asian	307 (1.5)	21 (1.0)
Black	1492 (7.2)	139 (6.4)
White	17 705 (86.0)	1929 (89.2)
Other^b^ or unknown	1077 (5.2)	74 (3.4)
Ethnicity		
Hispanic	644 (3.1)	57 (2.6)
Non-Hispanic	18 612 (90.4)	1989 (92.0)
Unknown	1325 (6.4)	117 (5.4)
Insurance type		
Commercial	13 138 (63.8)	1355 (62.6)
Medicaid	1056 (5.1)	19 (0.9)
Medicare	4733 (23.0)	683 (31.6)
Other or unknown	1654 (8.0)	106 (4.9)
RUCA^c^		
Metropolitan urbanized area	2069 (10.1)	1885 (87.1)
Large urban cluster	165 (0.8)	156 (7.2)
Small urban cluster	86 (0.4)	81 (3.7)
Rural	45 (0.2)	41 (1.9)
Unknown	18 216 (88.5)	0
Smoking status		
Never	6692 (32.5)	633 (29.3)
Not current, with history unknown	1235 (6.0)	104 (4.8)
Previous	6019 (29.2)	647 (29.9)
Current	3763 (18.3)	385 (17.8)
Unknown	2872 (14.0)	394 (18.2)
Charlson Comorbidity Index		
0	11 423 (55.5)	1222 (56.5)
1-2	5900 (28.7)	624 (28.8)
3-4	1687 (8.2)	190 (8.8)
≥5	778 (3.8)	68 (3.1)
Unknown	793 (3.9)	59 (2.7)

Abbreviations: EHR, electronic health record; RUCA, Rural-Urban Commuting Area.

^a^ Of 20 581 patients with EHR data, 2163 also had claims data. Unless otherwise indicated, data are expressed as No. (%) of patients.

^b^ Specific race categories were unavailable from the Optum Labs Data Warehouse.

^c^ Metropolitan urbanized area indicates population of 50 000 or greater; large urban cluster, population of 10 000 to 49 999; small urban cluster, population of 2500 to 9999; and rural, population of less than 2500.

### Measure Performance Rates

**Figure 2.**
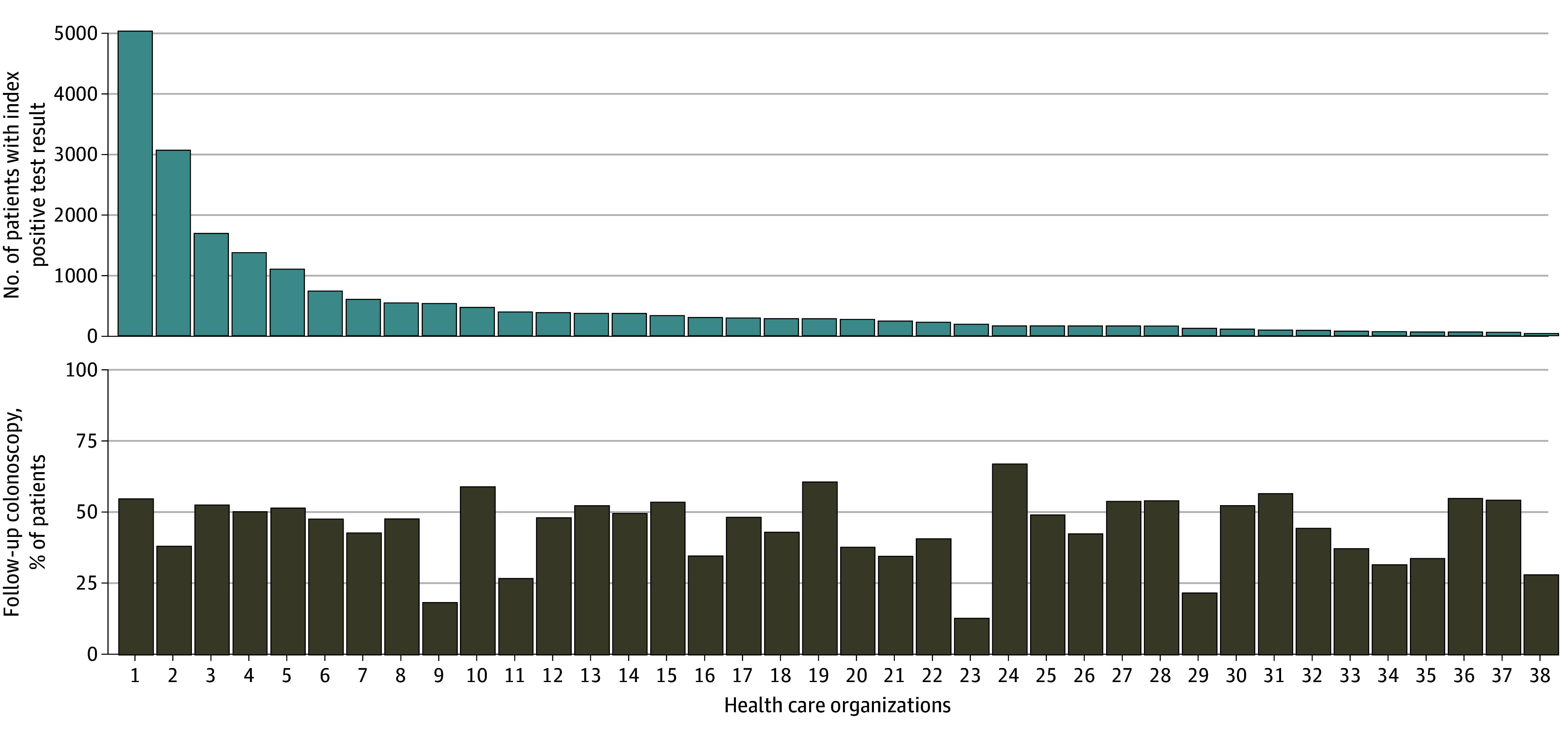
Measure Performance, by Health Care Organization and Volume of Tests With Positive Results, 2018 Follow-up colonoscopy within 180 days of abnormal stool-based test ranged from 13.1% to 66.9% (median, 47.9%) across 38 health care organizations.

**Figure 3.**
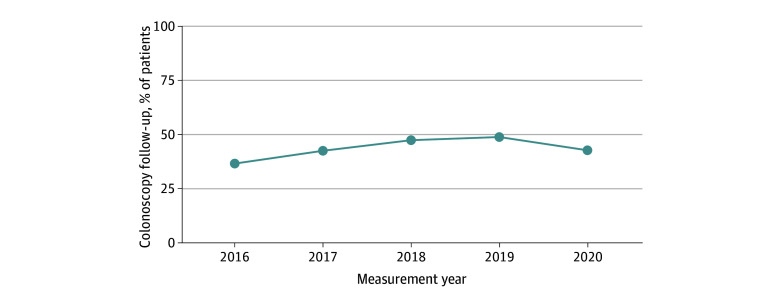
Measure Performance Over Time, 2016 to 2020

Across 38 HCOs, 20 581 patients returned an abnormal SBT result in 2018. Of these, 10 009 patients (48.6%) were men and 10 572 (51.4%) were women; mean (SD) age was 63.6 (7.1) years. In terms of race and ethnicity, 307 patients (1.5%) were Asian, 1492 (7.2%) were Black, 644 (3.1%) were Hispanic, 17 705 (86.0%) were White, and 1077 (5.2%) were of other or unknown race or ethnicity. A total of 13 138 patients (63.8%) had commercial insurance and 11 423 (55.5%) had no comorbidities (score of 0 on the Charlson Comorbidity Index^30^). The median number of patients was 274, ranging from 39 to 5012 per HCO. A median of 47.9% (IQR, 37.4%-53.2%) of patients with an abnormal SBT result received a follow-up colonoscopy within 180 days across HCOs, with a median follow-up time of 53 (IQR, 28-115) days. Performance ranged from 13.1% to 69.9% across organizations. Figure 2 shows the variation across organization by size of organization quantified by patient volumes. Variation in measure performance across HCOs is indicative of measure feasibility,^31^ as is the ability to move or change rates over time. Colonoscopy follow-up rates differed significantly across years (1-way repeated-measures ANOVA, *P* < .001) and increased by approximately 33% between 2016 and 2019, indicating the ability of HCOs to improve performance rates. Rates subsequently declined by 14% through 2020, presumably due to the COVID-19 pandemic (Figure 3).

For CRC screening, disparities by race and ethnicity are well established.^1^ In the current analysis, White patients had the highest follow-up rates in 2018 at 49.0% (95% CI, 48.2%-49.7%) compared with 37.1% (95% CI, 34.6%-39.5%) of Black and 38.4% (95% CI, 34.6%-42.1%) of Hispanic patients. Patients with Medicare or Medicaid insurance had follow-up rates of 49.2% (95% CI, 47.7%-50.6%) and 39.2% (95% CI, 36.3%-42.1%), respectively, compared with commercially insured patients at 50.9% (95% CI, 50.0%-51.7%). Differences across race, ethnicity, and insurance status were all significant (1-way ANOVA, *P* < .001).

### Reliability Testing

Reliability testing included testing across the 38 HCOs, 4 race categories (including other or unknown), 3 ethnicity categories (including unknown), and 5 measurement years (2016-2020). In the across-HCO comparison, a median of 94.5% (range, 74.3%-99.7%) of the variance in the measure was due to between-HCO differences. For the other categories examined, reliability rates were high at 94.2% (range, 79.5%-99.5%) across race, 93.1% (range, 86.7%-99.4%) across ethnicity, and 99.3% (range, 99.1%-99.4%) across measurement year (see Table 2). These results demonstrated good reliability, as values above 70% were considered sufficient to observe differences between HCOs or other stratifications.^29^

**Table 2.** Results From Reliability Testing

Group	No. of groups	No. of patients per group, median (range)	Reliability, median (range), %
Health care organization	38	274 (39-5012)	94.5 (74.3-99.7)
Race	4	6122 (1356-80 980)	94.2 (79.5-99.5)
Ethnicity	3	6231 (2901-85 448)	93.1 (86.7-99.4)
Measurement year	5	18 107 (15 563-24 283)	99.3 (99.1-99.4)

### Sensitivity Analysis

Two sensitivity analyses were conducted (eFigure 2 in Supplement 1). Among the subpopulation of patients with overlapping claims data (n = 2163), 59.9% (95% CI, 57.9%-62.0%) received a follow-up colonoscopy within 180 days compared with 51.3% (95% CI, 49.2%-53.4%) in this subpopulation when examining EHR data only. This demonstrates missed EHR capture of these procedures in approximately 14% of total patients who received a colonoscopy, a conservative estimate. Applying this to the full EHR population would increase the median measure performance rate to 54%.

A second sensitivity analysis evaluated measure performance comparing a 90- with a 180-day follow-up period. Compared with the median 47.9% follow-up rate within 180 days, the median follow-up rate within 90 days was 39.7% (IQR, 28.7%-44.0%) across HCOs. The difference represented 1524 tests, constituting 15.7% of total colonoscopies performed within 180 days.

### Feasibility Testing

Face validity was assessed through the engagement of 4 national CRC screening experts representing 2 HCOs, the American Cancer Society, the National Center for Quality Assurance, and the American Cancer Society National Colorectal Cancer Roundtable. Performance rates overall, over time, and stratified by patient characteristic, as well as reliability and feasibility testing results, were shared with the 4 advisors. Over 6 monthly 1-hour meetings, the advisors unanimously agreed to the validity of the results at face value, attesting that the results made sense and aligned with their expectations.

### Feasibility Field Testing Results

Feasibility field testing was conducted to determine the feasibility of collecting the required data elements for the measure within an EHR. At 3 HCOs, 3 different EHR vendors were represented in the feasibility field testing, including Epic Systems, Athenahealth, and Allscripts TouchWorks, to ensure compatibility across EHRs. Testing demonstrated that most data elements were feasible to collect, with 2 exceptions. First, the ability to ascertain patients receiving hospice or palliative care was not feasible 100% of the time. One HCO did not have a field in their EHR to capture hospice or palliative care, while the other 2 sites reported that this type of care is not consistently documented. Second, it was not universally feasible to capture data on inpatient stays and emergency department visits. The EHR systems of 2 HCOs did not capture data on inpatient stays and emergency department visits due to lack of an integrated system or a single EHR. The third HCO reported that these data were collected neither consistently nor in a standardized manner. All 3 testing sites reported the ability to identify diagnostic SBT results using *CPT* codes. The results of the feasibility field testing are provided in eTable 2 in Supplement 1.

## Discussion

Our findings suggest that a performance measure that evaluates completion of colonoscopy following an abnormal SBT result is feasible and reliable and has substantial variation across 38 health systems. The median HCO had a follow-up rate of 47.9% within 6 months, ranging from 13.1% to 69.9% across organizations. Due to well-established CRC screening disparities by race and ethnicity^1^ and a trend among quality measure developers and stewards to require stratification by proxy variables for potentially at-risk populations, we also examined disparities in colonoscopy follow-up. We observed lower rates among Black and Hispanic patients and those with Medicare or Medicaid insurance. The proportion of patients who received a follow-up colonoscopy within 6 months increased from 2016 through 2019, followed by a slight reduction in 2020, likely attributable to the effect of the COVID-19 pandemic on health systems and patients’ willingness to seek elective in-person care.^32,33^

Results from feasibility testing revealed that most data elements were feasible to capture in the EHR. Two data elements (hospital stay and emergency department visit) were not feasible to capture in some cases, but organizations reported the capacity to differentiate between screening and diagnostic SBT, and this could mitigate the need for these data elements. We therefore propose to exclude these as required data elements for the measure. The other issue that was identified, the inability to identify palliative or hospice care 100% of the time, was determined to have a negligible effect on measure rates because these patients rarely receive SBTs in practice. Therefore, receiving palliative care or hospice care should be maintained as an exclusion criterion.

A similar range of colonoscopy follow-up performance has been reported in previous studies.^8,16,18,20,34,35,36,37^ Mohl et al^20^ found a 6-month follow-up colonoscopy rate of 51.4%. Other studies^8,16,18,34,35,36,37^ have yielded rates ranging from 18% to 75%, with most well below the American Cancer Society National Colorectal Cancer Roundtable goal of 80% or higher in every US community. While a longer follow-up period might be considered based on data supporting an increased risk at 6 to 12 months, studies such as the one conducted by Mohl et al^20^ show that 90% of follow-up at 12 months is captured by 6 months. Since earlier detection is optimal and should be encouraged, the earliest feasible follow-up period was selected for the measure.

Stool-based tests are robust tools for CRC screening; however, their use only reflects part of the screening process. A timely follow-up colonoscopy following an abnormal SBT result is necessary to complete the screening process. To address this shortcoming, we have proposed a new measure to complement the existing HEDIS measure: the CRC screening completion measure. The measure is proposed as a health system measure, but where SBT results are available, the measure could also be considered for health plans. This measure is being tested in 20 health systems participating in a national CRC Screening Best Practices Learning Collaborative (2023-2025)^38^ and has been shown to be feasible to implement into practice. Ultimately, we hope to improve CRC screening rates and detection of CRC at earlier stages in efforts to reduce the burden of this disease on individuals and health care systems. While technically all noncolonoscopy tests—that is, SBTs, computed tomographic colonography, and flexible sigmoidoscopy—should be followed up with a colonoscopy, this measure is limited to SBTs due to the infrequent use of the other tests and the growing use of SBTs. In fact, more than 99% of CRC screening tests reported in the American Medical Group Association’s national CRC Screening Best Practices Learning Collaborative^38^ were colonoscopies or SBTs. Other noncolonoscopy tests could be considered for inclusion in this measure, although the impact on measure rates would be negligible.

### Strengths and Limitations

Strengths of this study include a large sample size of 38 diverse HCOs; our ability to test the reliability, variability, and face validity of the data over several years; and the demonstrated feasibility of collecting data elements in 3 independent HCOs. This study also has some limitations. Health systems may be limited in identifying colonoscopies that occurred outside their organizations. A sensitivity analysis found underestimates of the follow-up colonoscopy rate to be approximately 14%; however, because this rate was calculated using administrative claims data from a single payer, true underestimates of follow-up colonoscopies could differ among individual health systems. The clinical data used in this study were sourced from a sample of health systems that maintained a relationship with the provider of the source data (OLDW), which may introduce selection bias if they differed systematically from other US health systems.

## Conclusions

In this quality improvement study of 20 581 patients, the need for and feasibility of a new CRC screening follow-up measure were established. Complete screening (ie, initial screening plus follow-up of abnormal SBT results) for CRC can lead to earlier detection and better outcomes, improving overall population health. Use of SBTs may increase overall screening rates, but abnormal results must be followed up with a colonoscopy to diagnose CRC—ideally as soon as possible, but definitely within the 6 months after an abnormal test result. In fact, immediate follow-up should be emphasized due to the variation in stage at first cancer detection. The proposed CRC screening completion measure is a novel, innovative measure concept that builds on and addresses an important shortcoming in an existing measure and will help ensure complete screening for CRC. Measure performance was low enough, with substantial variation across health systems, to provide an opportunity for feedback and improvement. The measure was reliable, with variation between systems due to differences in performance, and was a feasible means of calculation and reporting using EHR data. Advancing this measure as a quality performance measure could significantly increase the early detection of CRC, thereby improving health and ultimately saving lives.

## Notes

Supplement 1. **eFigure 1.** Measure Schematic **eTable 1.** Feasibility Scorecard and Data Element Feasibility Domains **eFigure 2.** Sensitivity Analysis of Measure Performance, 90- vs 180-Day Follow-Up (n = 38) **eTable 2.** Results From Feasibility Field Testing

Supplement 2.
Data Sharing Statement
