# Supplementary material for: Development of a Follow-Up Measure to Ensure Complete Screening for Colorectal Cancer
Source: JAMA Netw Open. 2024 Mar 25;7(3):e242693. doi: 10.1001/jamanetworkopen.2024.2693 (PMC10964113; doi:10.1001/jamanetworkopen.2024.2693)
Supplement: Supplement 1. — eFigure 1. Measure Schematic eTable 1. Feasibility Scorecard and Data Element Feasibility Domains eFigure 2. Sensitivity Analysis of Measure Performance, 90- vs 180-Day Follow-Up (n = 38) eTable 2. Results From Feasibility Field Testing [file jamanetwopen-e242693-s001.pdf]

Supplementary Online Content

Ciemins EL, Mohl JT, Moreno CA, Colangelo F, Smith RA, Barton M. Development of a follow-up measure to ensure complete screening for colorectal cancer. *JAMA Netw Open*. 2024;7(3):e242693. doi:10.1001/jamanetworkopen.2024.2693

eFigure 1. Measure Schematic
eTable 1. Feasibility Scorecard and Data Element Feasibility Domains
eFigure 2. Sensitivity Analysis of Measure Performance, 90- vs 180-Day Follow-Up (n = 38)
eTable 2. Results From Feasibility Field Testing

This supplementary material has been provided by the authors to give readers additional information about their work.

© 2024 Ciemins EL et al. *JAMA Network Open*.

eFigure 1. Measure Schematic

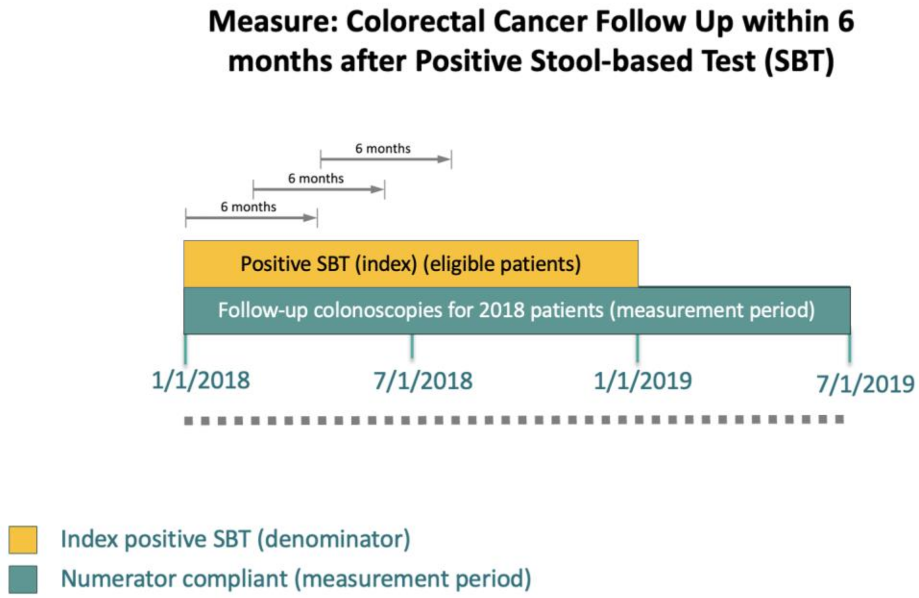

© 2024 Ciemins EL et al. *JAMA Network Open*.

eTable 1. Feasibility Scorecard and Data Element Feasibility Domains

Data Element Feasibility Domains		
**Definitions**	**Score**	**Examples**
**Availability - the extent to which the data are readily available in a structured format across EHR systems.**		
Data element exists in a structured format in this EHR.	**1**	Data are stored in tabular form with a field name and associated value.
Data element is not available in a structured format in this EHR.	**0**	Data element only exists in clinical notes or unstructured clinical documents.
**Accuracy - the extent to which the information contained in the data is correct.**		
Information is from authoritative source and/or is highly likely to be correct.	**1**	Lab results transmitted directly from the laboratory information system into the EHR, or data element included as a result of clinician assessment or interpretation. May also include patient-report data directly from an instrument.
Information may not be correct.	**0**	Check box that indicates medication reconciliation was performed, or self-report of a vaccination.
**Standards - the extent to which the data element is coded using a nationally accepted terminology standard (vocabulary) and mapped to the Quality Data model (QDM).**		
Data element is coded in a nationally accepted terminology standard or can be mapped to that terminology standard.	**1**	RXNORM, SNOMED, CPT, ICD10, HCPC, RxNorm, NDC, LOINC
Terminology standards for the data element are currently available, but not consistently coded to standard terminology in the EHR, or the EHR does not easily allow, or support, such coding.	**0**	
**Workflow - the extent to which capturing the data element impacts the typical workflow for that user.**		
The data element is routinely collected during clinical care and requires no, or limited, additional data entry from a clinician or other provider, and no EHR interface changes.	**1**	Lab values vital signs, referral orders, or problem list entry
Data element is not routinely collected during clinical care and additional time and effort are required to collect this data element without perceived benefit to care.	**0**	

© 2024 Ciemins EL et al. *JAMA Network Open*.

eFigure 2. Sensitivity analysis of Measure Performance, 90- vs 180-Day Follow-Up (n=38)

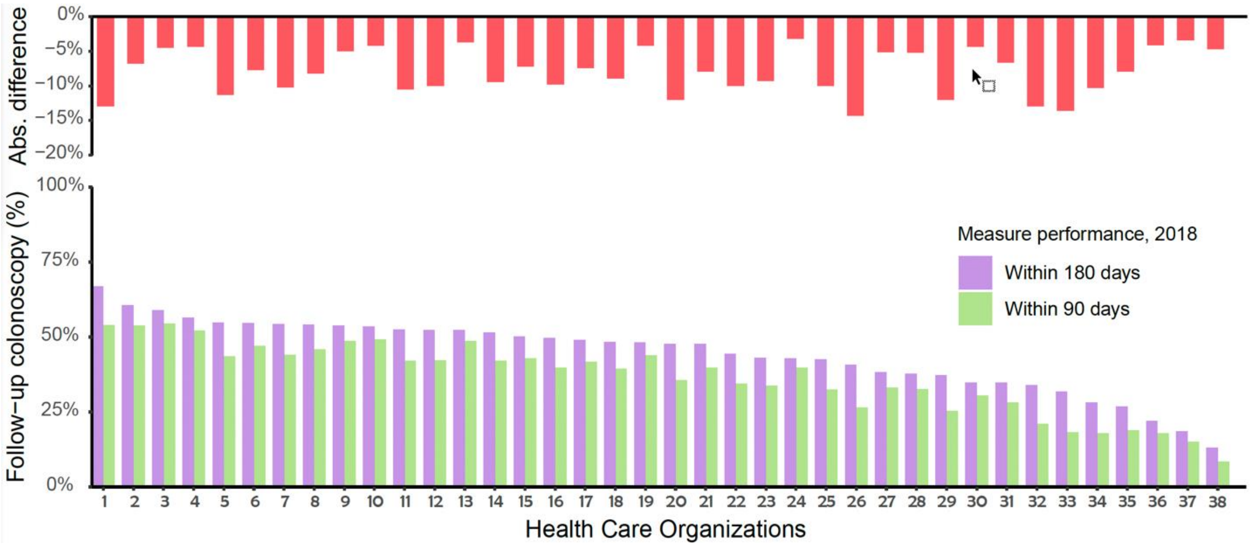

eFigure 2 Caption: Proportion of index cases followed up within 90 days and 180 days and ranged from 8.4% to 54.4% (median: 39.7%) and 13.1% to 66.9% (median: 47.9%), respectively. 6.7% of follow-up colonoscopies occurred between 90 and 180 days representing 15% of all colonoscopies performed. Differences in the rate of follow-up colonoscopies between 90 and 180 days ranged from 3.4% to 14.4% across the 38 HCOs.

© 2024 Ciemins EL et al. *JAMA Network Open*.

eTable 2. Results From Feasibility Field Testing

	EHR #1	Site #1			EHR #2	Site #2			EHR #3	Site #3		
**Data Element**	**DATA AVAILABILITY**	**DATA ACCURACY**	**DATA STANDARDS**	**WORKFLOW**	**DATA AVAILABILITY**	**DATA ACCURACY**	**DATA STANDARDS**	**WORKFLOW**	**DATA AVAILABILITY**	**DATA ACCURACY**	**DATA STANDARDS**	**WORKFLOW**
Patient age	1	1	1	1	1	1	1	1	1	1	1	1
Date and result of CRC screening SBT	1	1	1	1	1	1	1	1	1	1	1	1
Encounter: Identification of prior CRC diagnosis	1	1	1	1	1	1	1	1	1	1	1	1
Encounter: Identification of prior total colectomy	1	1	1	1	1	1	1	1	1	1	1	1
Encounter: Initiation of hospice or palliative care within 12 months of positive SBT	0	0	0	0	1	0	0	0	1	0	1	1
Encounter: Identification of inpatient visit within 14 days prior to or following positive SBT	0	0	0	0	1	1	0	1	0	0	0	0
Encounter: Identification of ER visit within 14 days prior to or following positive SBT	0	0	0	0	1	1	0	1	0	0	0	0
Encounter: Identification of diagnostic SBT per CPT code within 3 days prior to positive test result	1	1	1	1	1	1	1	1	*	*	*	*
Follow-up colonoscopy date	1	1	1	1	1	1	1	1	1	1	1	1
Race	1	1	1	1	1	1	1	1	1	1	1	1
Ethnicity	1	1	1	1	1	1	1	1	1	1	1	1
**SUMMARY**												
Data Elements Scoring 0 within Domain	3	3	3	3	0	1	3	1	2	3	2	2
Total data elements	11	11	11	11	11	11	11	11	10	10	10	10
% of data elements requiring review within domain	27%	27%	27%	27%	0%	9%	27%	9%	20%	30%	20%	20%

© 2024 Ciemins EL et al. *JAMA Network Open*.
