# Supplementary material for: Development of a Follow-Up Measure to Ensure Complete Screening for Colorectal Cancer
Source: JAMA Netw Open. 2024 Mar 25;7(3):e242693. doi: 10.1001/jamanetworkopen.2024.2693 (PMC10964113; doi:10.1001/jamanetworkopen.2024.2693)
Supplement: Supplement 2. — Data Sharing Statement [file jamanetwopen-e242693-s002.pdf]

Data Sharing Statement

Ciemins. Development of a Follow-Up Measure to Ensure Complete Screening for Colorectal Cancer. *JAMA Netw Open*. Published March 25, 2024.
doi:10.1001/jamanetworkopen.2024.2693

Data
Data available: No

Additional Information
Explanation for why data not available: The data were sourced from a de-identified data asset, the Optum Labs Data Warehouse. They do not make their data available at the patient level. Other data collected during the measure testing activities can be shared.
